# Research on the influence of maker spirit on knowledge workers’ innovative behavior

**DOI:** 10.3389/fpsyg.2023.1182001

**Published:** 2023-12-07

**Authors:** Quanxiang Xue, Can Liu, Min Zhao, Hui Jin

**Affiliations:** ^1^School of Economics and Management, Jiangsu University of Science and Technology, Zhenjiang, China; ^2^Business School, Hohai University, Nanjing, China

**Keywords:** maker spirit, knowledge workers, job crafting, superiors’ developmental feedback, innovative behavior

## Abstract

Motivating the innovative behavior of knowledge workers with the “maker spirit” is important for enhancing innovation efficiency. Based on the unique “maker spirit” embodied in knowledge workers in Chinese, this study comprehensively considers elements of job crafting and superiors’ developmental feedback, and uses questionnaire survey method and mathematical statistical analysis method to explore the relationship between the maker spirit and the innovative behavior of knowledge workers in order to provide theoretical support for further promoting the development of social innovation. The results of the study show that the spirit of innovation, sharing, practice, and entrepreneurship in the maker spirit all have a positive contribution to innovative behavior; job crafting mediates between the spirit of innovation, sharing, practice, entrepreneurship, and innovative behavior; and superiors’ developmental feedback plays a positive moderating role between the spirit of innovation, sharing, practice, entrepreneurship, and job crafting.

## Introduction

In the book “Milestones of Tomorrow” published in 1959, Drucker first proposed the concept of knowledge workers (Drucker, 1959), and pointed out in “Management Challenges in the 21st Century”: in the context of global innovation, in view of the basic reality that knowledge workers gradually replace manual workers and become the main production force of enterprises, it is of great significance to improve the ability and productivity of knowledge workers to promote knowledge management (Fraser and Simkins, 2016). Since the beginning of the 21st century, with the increasingly obvious characteristics of network and informatization of the global economy, countries have generally formed a consensus in the process of economic development that innovation is the driving force to promote economic development and improve the level of productivity (Sha et al., 2022). As the main carrier of innovation activities, how to stimulate the innovation behavior of knowledge workers, enhance their innovation level and achieve efficient innovation output has become a hot topic of concern for many countries, governments, and scholars (Su et al., 2021). As the link between innovation consciousness and innovation achievements, the innovative behavior of knowledge workers can effectively promote the transformation of their innovation consciousness into innovation achievements, and bring considerable economic benefits to society, enterprises, and individuals. Specifically, knowledge workers’ innovative behavior is not only an important indicator of their efficiency, but also a necessary requirement for the production of innovative achievements. In recent years, the output of technological achievements such as the synthesis of starch from carbon dioxide, quantum computers, and artificial intelligence chips have all demonstrated that knowledge workers can use their efficient innovative behavior as an engine to promote the advancement of science and technology.

As an important group of knowledge workers, makers have a long history of development. In the course of its development history, maker has actively carried out technology research based on platforms such as makerspaces, technology incubators, and business incubators, and their innovative behavior has had an important impact on China’s manufacturing, economy, culture, and other fields (Mcgrath and Guglielmo, 2015). In 2015, Chinese Premier Li Keqiang also proposed: “Makers fully demonstrate the vitality of mass entrepreneurship and innovation, and this vitality and creativity will become the unquenchable engine of China’s economic growth in the future.” In recent years, thanks to the rapid development of technologies such as 3D printing, 3D scanning, and CNC laser etching, it has become much less difficult for makers to transform their ideas into innovative products. This not only further promotes the development of the maker group, but also stimulates the innovative behavior and vitality of knowledge workers represented by makers. As China’s economy enters a state of high-quality development driven by innovation, the surge in demand for product innovation, business model innovation, and service innovation will inevitably lead to the concentration of makers in makerspaces. In the process, the sharing of knowledge, the pooling of wisdom and co-creation among creators promote the formation of the group’s values – the Maker Spirit. As a unique innovation element, the maker spirit can promote the sharing of knowledge, the circulation of resources and mutual recognition among makers, energize the innovative behavior of knowledge workers (Schroder and Arnaud, 2011) and is the source and driving force for the sustainable development of maker groups (Anamian et al., 2016; Martin and Upham, 2016). However, most Chinese governments and administrators are not fully aware of the importance of maker spirit, and the research on maker spirit still remains at the stage of simple qualitative analysis, failing to explore the inner connection between maker spirit and innovation behavior of knowledge workers, which not only greatly inhibits the driving effect of maker spirit on innovation behavior of knowledge workers, but also hinders the sustainable development of Chinese maker community and technology level. In view of this, in order to clarify the effect mechanism of maker spirit on knowledge workers’ innovation behavior, this article combines existing literature and theories, introduces factors such as job crafting and superiors’ developmental feedback, and uses questionnaires and mathematical statistical analysis methods to explore the relationship between the maker spirit and innovative behaviors of knowledge workers. In order to provide strategic suggestions and empirical references for encouraging knowledge workers to carry out innovative behaviors, stimulating innovation, and optimizing the management of knowledge workers under the background of the maker spirit as the core.

## Literature review and research hypothesis

From the perspective of the object in this study, the existing literature can be divided into two main categories of creative spirit and innovative behavior.

### Maker spirit

The maker spirit is the core of the maker movement (Kruger et al., 2019), the intrinsic motivation to gather makers and entrepreneurial resources, and the important force to push makers forward continuously. From a micro perspective, the maker spirit is the driving force that exists in the maker’s heart and affects its behavior. It is the key to the transformation of entrepreneurial ideas from technology-oriented to demand-oriented and entrepreneurial activities from internal to open, which is important for shaping innovation consciousness and improving innovation performance of makers (Ross and Michael, 1989). From the macro perspective, the maker spirit plays an important role in creating an innovative and entrepreneurial atmosphere and activating innovative and entrepreneurial activities, and is seen as an important engine for achieving a new round of economy (Lindtner and Li, 2012).

The formation of a maker spirit is a complex process. Muramatsu et al. (2013) argued that the factors influencing the formation of maker spirit are divided into endogenous and exogenous factors. That is, endogenous factors represented by subjective perception, personal interest and prior experience and external factors represented by family education, school education, and social network. Paulin (2013) believed that the generation of maker spirit is an evolutionary process. The interaction and co-evolution of internal and external factors are the driving force behind the development of the maker spirit. Blumentritt et al. (2005) believed that the cultivation of the maker spirit should be carried out from four dimensions: system, environment, values, and economy. Hovhannisian (2004) believed that the formation of creativity is closely related to corporate, national, and professional cultures, and the evolution of the aforementioned cultures enriches the connotation of the maker spirit and provides nourishment for its sustainable development.

The maturation and promotion of the maker spirit has stimulated the vitality of innovation in the whole society. It is of great significance for the development of various innovation groups. Based on the theory of entrepreneurial culture, Kubica and Szynkiewicz (2006) suggested that the spirit of entrepreneurship can strengthen the practical awareness of entrepreneurship among young people and motivate them to initiate entrepreneurial activities. Chanal and Caron (2010) found that maker spirit is conducive to enhancing the openness of business models and facilitating knowledge sharing, which in turn reduces the R&D costs of corporate innovation. Taking the Central and Eastern European University as the research object, Boyle and Thomas (2007) found that the maker spirit has an important role in promoting the motivation of students’ entrepreneurial behavior and developing innovative thinking.

In summary, although different scholars define maker spirit differently, it mainly revolves around the four elements of innovation, sharing, practice, and entrepreneurship. At the same time, the existing literature shows that the maker spirit has a significant impact on various innovative activities of the subject. Therefore, based on the combination of related literature, this article defines maker spirit as the spirit of innovation, sharing, practice, and entrepreneurship shown by makers in a free environment, and holds that it can influence innovative behavior.

### Innovative behavior

Innovative behavior is the main source for innovative subjects to gain innovative momentum and maintain competitiveness (Chollet et al., 2012). In terms of the conceptual content of innovative behavior, innovative behavior can be regarded as a method of innovation subjects in their work, which mainly includes the generation, realization, promotion, and improvement of innovative ideas (Wheeler et al., 2016). Kanter (1996) argued that innovative behavior in the initial formation stage is simply the recognition of a problem and the generation of innovative ideas. Subsequently, after a series of complex processes, innovative behavior is transformed into a new product or service for the firm.

Given the importance of innovative behavior for the development of social innovation, the existing research literature has conducted in-depth studies on its influencing factors. Based on structural equation modeling, Seop and Li (2018) empirically found that there are positive relationships between transformational leadership and organizational embeddedness, and organizational embeddedness and innovative behavior, and proposed that the performance of leaders has a significant impact on the innovative behavior of employees, especially in terms of encouraging employees to proactively embed themselves in their organizations. Wan et al. (2022) argued that there is a significant crowding-out effect of tax incentives on the innovation behavior of Chinese high-tech industries, and economic policy uncertainty has a negative moderating effect on the relationship between tax incentives and innovation behavior. Mitchell and Lee (2001) first introduced the term of job embeddedness and found that highly job embedded people who experience shocks have fewer plans about leaving than people who are low on embeddedness and who experience a shock. This stable employee relationship helps employees concentrate on their work and carry out creative activities to improve work efficiency. Teece (1992) found that in a highly competitive environment, the strategic alliances-constellations of bilateral agreements among firms facilitates the implementation of innovative behaviors. West (2001) found that personal traits such as self-confidence, intelligence, and perseverance have a significant impact on an individual’s innovative behavior. In work teams, clear goals, high levels of engagement, commitment to excellence, and have a facilitating effect on employee innovative behavior. Dreu and West (2001) found that enhance employees’ participation in decision-making is conducive to stimulating their innovative enthusiasm, while different opinions stimulate employees’ creativity and divergent thinking, helping them to carry out innovative behaviors.

In general, the existing literature has extensively studied maker spirit and innovative behavior. However, few scholars have integrated the two into a unified analytical framework to comprehensively analyzed the influence mechanism of maker spirit on innovation behavior. To fill this research gap, this article constructed an empirical model based on the introduction of variables such as job crafting and superiors’ developmental feedback to investigate the impact of maker spirit on the knowledge workers’ innovative behavior. It is intended to provide strategic suggestions and empirical references for stimulating the innovation behavior of knowledge workers, improving the efficiency of innovation, and promoting the development of China’s innovation economy under the background of the maker spirit as the core.

### Research hypothesis

#### Maker spirit and knowledge workers’ innovative behavior

Social capital theory states that social capital is the ability of an individual to derive benefits from social networks. These benefits generally include privileged access to knowledge and information, access to open-source software, access to new business advantages and opportunities, etc. From the perspective of creators, maker spirit as social capital rooted in relationships of trust, collaboration, and cooperation among makers can bring profit and utility to creators engaged in innovative work and promote innovative behavior. Specifically, firstly, knowledge workers can use maker spirit to strengthen communication and cooperation with different individuals in the innovation process, and to obtain the knowledge, information and technology needed for innovation. Secondly, the innovation process of knowledge workers is often characterized by greater risk and uncertainty. Maker spirit provides a platform and opportunity for innovation cooperation between different knowledge workers inside and outside the organization. Non-technical factors such as interpersonal relationships and cultural atmosphere brought by strong maker spirit have an important impact on coping with uncertainty in innovation, promoting cooperation, strengthening synergy, and facilitating the success of innovation. Thirdly, the maker spirit as social capital is uniquely regional, and innovation networks formed on the basis of regional social capital are generally more stable and have very unique regional qualities that are difficult to be emulated and weakened by other regions. This advantage can greatly promote the development of innovation in the region, and it is easy to form regional advantages and characteristics. Fourthly, relying on the maker spirit of social networks, knowledge workers, while acquiring social capital, are also actively integrated into the regional innovation network formed by the social structure. Based on the above mentioned theoretical and literature achievements, this study considered the relationship between the maker spirit and knowledge workers’ innovation behavior in terms of four aspects: innovation spirit, sharing spirit, practical spirit, and entrepreneurial spirit.

Innovation spirit is a proactive, creative, risk-taking, open-minded, resilient and market-oriented mindset, and attitude. It motivates individuals and organizations to explore new ideas and solutions. The innovation spirit helps to drive social, scientific, technological, and economic progress. It creates new value and improves the quality of life. It is a critical element for success and sustainable development. When knowledge workers show enthusiasm for innovative work, it will naturally be reflected in their expression, language, and actions (Fei and Zhang, 2017). Knowledge workers will therefore unconsciously carry out innovation behaviors (Sun et al., 2014).

Sharing spirit is a social value that emphasizes the willingness of people to share resources, knowledge, time and care in order to promote social solidarity, cooperation and common prosperity. In their work processes, knowledge workers can share experiences with colleagues, promote knowledge transfer within the enterprise, and provide knowledge sources for innovation activities (Chang and Hung, 2021). The sharing spirit of knowledge workers thus plays an important role in the innovation behavior of knowledge workers (Taa et al., 2020).

Practical spirit is an attitude that focuses on action and practical experience, emphasizing the ability to put ideas into practice, active problem-solving and continuous learning. It encourages people to practice, gain experience, adapt to change, and improve through feedback. The practice of knowledge workers is social and refers to all the actions of people to transform and explore the real world. Knowledge employees constantly cultivate their practical abilities to identify problems, connect theory with practice, achieve breakthroughs, accumulate experience, develop themselves, and ultimately promote their own innovation behavior (Lin, 2007; Rui and Cheng, 2019).

Entrepreneurial spirit is a proactive, creative, and resilient attitude that emphasizes the ability to create new ventures or undertake innovative projects. It includes the qualities of being willing to take risks, seek new opportunities, overcome challenges and pursue success. Entrepreneurial spirit encourages individuals or teams to create new value, drive economic growth, create jobs, and solve social problems. This spirit is particularly important among start-ups and entrepreneurs, but is also important for the career development of individuals and the competitiveness of organizations. Previous studies have characterized entrepreneurship as a process of seeking opportunities, creating value, and seeking appreciation, emphasizing the entrepreneurial purpose in innovative ways through the efforts of individuals or groups (Siqueira et al., 2021). This definition implies that entrepreneurship is an innovative behavior, and the entrepreneurial process of knowledge workers therefore falls within the scope of knowledge workers’ innovation behavior (Shurbagi and Zahari, 2014).

Based on the above, the following hypotheses are proposed:

H1a: Innovation spirit has a positive effect on knowledge workers’ innovation behavior.

H1b: Sharing spirit has a positive effect on knowledge workers’ innovative behavior.

H1c: Practical spirit has a positive effect on knowledge workers’ innovative behavior.

H1d: Entrepreneurial spirit has a positive effect on knowledge workers’ innovative behavior.

#### Maker spirit and job crafting

The maker spirit will stimulate knowledge workers’ demand for knowledge, technology and other resources, so that knowledge workers can continuously improve themselves in the process of pursuing knowledge accumulation (Rui and Ling, 2008). At the same time, for knowledge workers, the maker spirit can promote them to undertake more challenging tasks and acquire technical capabilities, thereby motivating them to break through themselves and realize their own work crafting (Zhang and Wei, 2010).

Being innovative means that knowledge workers are willing to break new ground and take on challenging work. Firstly, individuals who are highly motivated to innovate are more likely to engage in extensive job crafting activities in order to fulfill their quest for a sense of competence. Secondly, if individuals are not innovative, it means that they lack the motivation to innovate and a sense of competence, and will not take the initiative to reshape their work. Finally, in the process, the spirit of innovation can provide direction and strategic guidance for job crafting (Yoo, 1987).

The spirit of sharing can effectively avoid duplication of effort and promote the interchange and sharing of resources between knowledge workers. When knowledge workers with the spirit of sharing gather together, they can share their experience with each other to improve work efficiency, so that they can concentrate on improving their skills and acquiring the resources needed for development, and ultimately create value for the enterprise (Sharabi et al., 2019). As a result, knowledge workers with a sharing spirit will adopt more job crafting behaviors.

In the context of resource scarcity, knowledge workers with practical spirit tend to actively seek out various resources before others, and are more able to seize the opportunity to find the resources needed to complete the work. At the same time, the spirit of practice can help knowledge workers to respond quickly to problems in their work and take appropriate measures for job crafting (Friedman et al., 2000). Thus, a spirit of practice can facilitate job reshaping by knowledge workers. Thus, a spirit of practice can facilitate job crafting by knowledge workers.

Entrepreneurial people are better at seizing opportunities and will act more quickly to carry out their work rather than wait and consider. At the same time, entrepreneurial knowledge workers are more proactive in using existing knowledge to uncover problems in their work. This means that entrepreneurial knowledge workers are better equipped to identify problems and solve them, and to respond to them by actively reinventing their work (Kuschminder et al., 2013). Therefore, entrepreneurship is conducive to the job crafting by knowledge workers.

Based on the above, the following hypotheses are proposed:

H2a: Innovation spirit has a positive effect on knowledge workers’ job crafting.

H2b: Sharing spirit has a positive effect on knowledge workers’ job crafting.

H2c: Practical spirit has a positive effect on knowledge workers’ job crafting.

H2d: Entrepreneurial spirit has a positive effect on knowledge workers’ job crafting.

#### Job crafting and knowledge workers’ innovative behavior

Job crafting refers to a series of self-imposed positive behaviors that change the boundaries of an employee’s work tasks and relationships in order to align his or her interests, motivations, and passions with the work. The focus of this approach is to use the power of active behavior to build a bridge between personal career and social meaning, and to gain a stronger sense of meaning and satisfaction by adjusting certain aspects of work. Knowledge workers’ innovation behavior is a process of identifying problems, creating new ideas, and putting those ideas into practice. Career-construction theory suggests that a person’s career develops based on his or her initiative, and adopting behaviors to adapt to dynamic environments can often result in career success (Wang, 2019). In the context of this study, job crafting involves actively constructing work based according to need and adapting to dynamic environments based on existing conditions. Knowledge workers with high-level job-crafting ability will take the initiative to improve their abilities so they can more easily adapt to the work environment and be more creative.

Increasing structural work resources (e.g., mastering new skills, improving professionalism, and increasing resource diversity) lays a strong foundation for knowledge workers’ innovative behavior. Increasing social work resources (e.g., seeking social support and increasing feedback from superiors) can improve the scope and quality of communication, which is conducive to obtaining more resources; this in turn will promote knowledge workers’ innovation behaviors. By exploring more challenging work opportunities, knowledge workers can further tap their potential, outperform themselves, and flexibly use resources for innovation (Zhan and Hu, 2021). Reducing work obstacles (e.g., reducing time-consuming tasks and counterproductive psychological pressures) helps knowledge workers deal with external pressures, control their emotions, and establish a safe environment for innovation (Li et al., 2020).

Based on the above, the following is proposed:

H3: Job crafting has a positive effect on knowledge workers’ innovative behavior.

#### Mediating role of job crafting

Resource conservation theory suggests that people with more resources are less susceptible to the negative effects of resource loss and are more capable of acquiring resources, while people with fewer resources are more susceptible to the negative effects of resource loss and find it difficult to acquire additional resources, and this can have a negative impact on an individual’s psychological state and behavioral patterns. Job crafting, as an individual’s bottom-up behavior to change the way of work, is also the original spontaneous acquisition of resources, which plays an important role in promoting the individual’s resource integration, enhancing the ability of resource acquisition, accelerating the spiral of resource acquisition, and promoting the individual’s implementation of innovative behaviors. Innovative activities are characterized by low marginal costs and high fixed costs. In the process of individual innovation, the more resources they enjoy, use and integrate, the lower the fixed cost of innovation, and the more inclined they are to implement innovative behavior. Based on JD-R theory, through job crafting, individuals can obtain more social resources, and then harvest more knowledge and information to optimize innovative thinking, enhance innovative ideas, identify innovative opportunities, and implement innovative behaviors; through job crafting, individuals can obtain more structural job resources, and then integrate the work tasks and better match them with their own professional skills and job requirements, improve the individual’s perceptual control over the innovation process, and enhance their innovative willingness and behaviors.

Specifically, the more innovative knowledge workers are, the more motivated they are to innovate, and the more they will take the initiative to meet challenging work requirements and perform job crafting, thereby gaining a sense of achievement (Lee and Roh, 2020). The higher the sharing spirit, the more knowledge workers can gain from sharing with other knowledge workers. This helps to avoid unnecessary mistakes and labor so that knowledge workers can focus on improving their skills and enhancing their own job crafting (Bemelmans et al., 2013). The more hands-on knowledge workers are, the more proactive they are in acquiring resources, reacting to problems, and redesigning their work tasks, thus facilitating job crafting (Yu and Guo, 2016). The more entrepreneurial spirit knowledge workers have, the more proactive they are in using their knowledge to innovatively design work tasks, thus contributing to job crafting (Wang et al., 2021). Through job crafting, knowledge workers take the initiative to improve their abilities, thus equipping themselves with better skills, adapting more easily to work environments, and generating more creative behaviors.

Based on the above, the following hypotheses are proposed:

H4a: Job crafting mediates the relationship between innovation spirit and knowledge workers’ innovative behavior.

H4b: Job crafting mediates the relationship between sharing spirit and knowledge workers’ innovative behavior.

H4c: Job crafting mediates the relationship between practical spirit and knowledge workers’ innovative behavior.

H4d: Job crafting mediates the relationship between entrepreneurial spirit and knowledge workers’ innovative behavior.

#### Moderation of superiors’ developmental feedback

Trait activation theory suggests that the efficacy of individual traits (e.g., personality, motivation, and cultural values) on individual behavior is based on the context, and that the effectiveness of individual traits on individual behavior depends on whether the context can provide elements to “activate” individual traits. Superiors’ developmental feedback, as positive feedback from supervisors to their subordinates, conveys positive signals to subordinates that their supervisors are concerned about them and recognize them. Therefore, with positive encouragement and motivation from supervisors, knowledge workers with a high level of creativity will be more motivated to reinvent their work. From the perspective of future learning, the superiors’ developmental feedback releases the leaders’ expectations for knowledge workers’ continuous learning, self-improvement, and change and growth, so in order to respond to the encouragement and advocacy of superiors, knowledge workers with a high level of maker spirit will be more likely to balance the relationship between work tasks and resources through job crafting, and take proactive behavior to achieve self-growth and improvement through their own learning. In summary, superiors’ developmental feedback has a moderating effect between creativity and job crafting, which can further strengthen knowledge workers’ motivation to reinvent their jobs, motivate employees to respond to leadership initiatives through job crafting, and provide direction for knowledge workers’ job crafting.

Leaders use developmental feedback to encourage positive behavior by subordinates, give them useful information, and promote a passion for work. When superiors’ developmental feedback is high, knowledge workers with innovation spirit will identify with the superiors and share their goals, which will reduce the risks associated with job crafting and improve the effect of innovation spirit on job crafting (Su et al., 2019). Meanwhile, when superiors’ developmental feedback is low, knowledge workers do not identify with their superiors, their innovation willingness is reduced, and job-crafting activities are not carried out normally (Wrzesniewski et al., 2013). Thus, the promotion effect of innovation spirit on job crafting is greatly reduced.

When superiors’ developmental feedback is high, knowledge workers with sharing spirit can obtain more effective resources, which will support their job-crafting activities and thereby improve the effect of sharing spirit on job crafting (Zhao et al., 2021). Meanwhile, when superiors’ developmental feedback is low, knowledge workers receive fewer resources; thus, the resources needed for job crafting are scarce. Therefore, the promotion effect of sharing spirit on job crafting will be greatly reduced (Lin et al., 2010).

According to the principle of reciprocity in social-exchange theory, when one party provides resources or opportunities, the other party will reciprocate in its own way. When superiors’ developmental feedback is high, knowledge workers with practical spirit will seek to reciprocate superiors with good work performance. Thus, knowledge workers will adjust their work through job crafting, thereby improving the effect of practical spirit on job crafting (Lee, 2020). Meanwhile, when superiors’ developmental feedback is low, knowledge workers are less inclined to reciprocate through good performance, which will reduce job-crafting behavior. Thus, the promotion effect of practical spirit on job crafting will be greatly reduced (Li, 2021).

When superiors’ developmental feedback is high, knowledge workers are encouraged, and their entrepreneurial spirit is stronger. Knowledge workers will take the initiative to acquire professional skills and expand their social circle; then, their job-crafting behavior will be promoted, and the effect of entrepreneurial spirit on job crafting will be improved (Hussein and Yesiltas, 2020). Meanwhile, when superiors’ developmental feedback is low, knowledge workers may lack identification with superiors, which can lead to organizational discord and counterproductive outcomes (Huang et al., 2021). Thus, the promotion effect of entrepreneurial spirit on job crafting will be greatly reduced.

Based on the above analyses, the following are proposed:

H5a: Superiors’ developmental feedback positively moderates the relationship between innovation spirit and job crafting; that is, the stronger the superiors’ developmental feedback, the stronger the effect of innovation spirit on job crafting.

H5b: Superiors’ developmental feedback positively moderates the relationship between sharing spirit and job crafting; that is, the stronger the superiors’ developmental feedback, the stronger the effect of sharing spirit on job crafting.

H5c: Superiors’ developmental feedback positively moderates the relationship between practical spirit and job crafting; that is, the stronger the superiors’ developmental feedback, the stronger the effect of practical spirit on job crafting.

H5d: Superiors’ developmental feedback positively moderates the relationship between entrepreneurial spirit and job crafting; that is, the stronger the superiors’ developmental feedback, the stronger the effect of entrepreneurial spirit on job crafting.

#### Moderated mediation effect of superiors’ developmental feedback

Finally, we proposed that superiors’ developmental feedback moderates not only the relationship between maker spirit and job crafting, but also the mediating process by which job crafting connects maker spirit and innovative behavior. Synthesizing H4a, H4b, H4c, H4d, H5a, H5b, H5c, and H5d, we suggested that the indirect effect of innovation spirit, sharing spirit, practice spirit, and entrepreneurship spirit on innovation behavior through job crafting is enhanced when superiors’ developmental feedback is high.

Based on those, the following are proposed:

H6a: Superiors’ developmental feedback moderates the indirect effect of innovation spirit through job crafting on innovative behavior, and the positive effect of innovation spirit through job crafting on innovative behavior is augmented when superiors’ developmental feedback is stronger.

H6b: Superiors’ developmental feedback moderates the indirect effect of sharing spirit through job crafting on innovative behavior, and the positive effect of sharing spirit through job crafting on innovative behavior is augmented when superiors’ developmental feedback is stronger.

H6c: Superiors’ developmental feedback moderates the indirect effect of practical spirit through job crafting on innovative behavior, and the positive effect of practical spirit through job crafting on innovative behavior is augmented when superiors’ developmental feedback is stronger.

H6d: Superiors’ developmental feedback moderates the indirect effect of entrepreneurial spirit through job crafting on innovative behavior, and the positive effect of entrepreneurial spirit through job crafting on innovative behavior is augmented when superiors’ developmental feedback is stronger.

**FIGURE 1:**
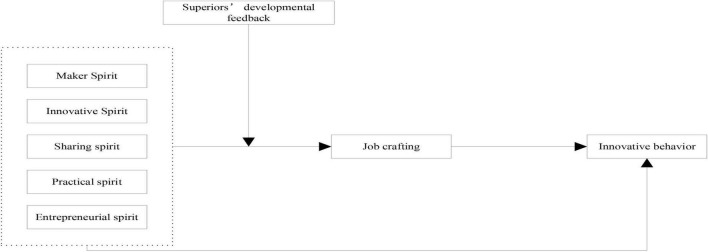
Theoretical model.

Figure 1 depicts the theoretical model of this study.

## Research methods

### Sample selection

Knowledge workers are the research object of this study. To ensure standardization, subjects were selected based on the definition of knowledge workers: educated people with professional and technical skills at the college level or above. The development of the maker spirit and the innovative activities of knowledge workers require a great deal of resources, and cities, as important carriers of regional resource convergence, can provide relatively complete supporting facilities and working environment for knowledge workers, so knowledge workers mainly gather in cities (Duggleby et al., 2004). As the most mature metropolitan area in China, the Yangtze River Delta region is at the forefront of all regional innovation indicators in China (Yin et al., 2022). Therefore, the selection of Shanghai, Jiangsu, and Zhejiang can fully guarantee the scientificity, validity, and representativeness of sample. In summary, on the basis of eliminating positions with strict regulations and less suitable for innovation, the sample was mainly selected from knowledge workers working in technology, R&D, operations, marketing, training, and purchasing in Jiangsu, Zhejiang, and Shanghai.

### Data sources

Data were collected using field research and questionnaires by post. Specifically, we conducted field visits and mailed questionnaires on knowledge workers with maker spirit working in local universities, scientific research institutions, technology-based companies, information consulting firms, and service enterprises to obtain sample data. A total of 600 questionnaires were distributed, and 528 were collected. After excluding invalid questionnaires (e.g., those with missing items or inconsistencies), 447 valid questionnaires were obtained. The sample was selected in consideration of the nature of the enterprise and its industry, and it covered different age groups, genders, education levels, positions, and years of employment. Thus, the sample had good representativeness. Among respondents, 51.91% were male and 48.09% were female. Table 1 shows the distribution of sample characteristics.

**TABLE 1:** Descriptive statistics of the sample.

Item	Index	Quantity	Ratio (%)	Item	Index	Quantity	Ratio (%)
Age	25 or below	53	11.91	Position	Lower-level workers	290	64.72
	26 30	320	71.46		Lower management	83	18.65
	31 35	37	8.31		Middle management	40	8.99
	36 40	9	2.02		Advanced management	34	7.64
	Over 41	28	6.30	Ownership	State enterprise	116	26.07
Education	College	54	12.13		Private enterprise	130	29.21
	Bachelor	360	80.45		Joint venture	24	5.39
	Master	23	5.17		Foreign-funded enterprise	19	4.27
	Doctor	10	2.25		Other business	158	35.06
Working years	3 years or less	298	66.51	Industry	E-communication	73	16.40
	3 5 years	50	11.24		Mechanical manufacturing	43	9.66
	5 10 years	50	11.24		Biopharmaceuticals	35	7.87
	Over 10 years	49	11.01		Chemical foods	39	8.77
					Other industries	257	57.30

### Variable selection and measures

Some variables in this study were measured use mature scales. Five-point Likert scales were used for all measures except the control variables; “1” indicated “complete disagreement,” and “5” indicated “complete agreement.” Regarding scale translation, back translation was used to ensure consistency between the meanings of the original English scales and the Chinese scales. Details about the scales are provided below.

### Explained variable: knowledge workers’ innovation behavior

Derived from Kafashpoor et al. (2013), this scale has been widely used to measure knowledge workers’ innovation behavior. There are six question items (Table A1).

### Explanatory variable: maker spirit

The scale for innovation spirit was derived from Robertson and Chetty (2000), and the scales for sharing spirit and practical spirit were derived from Zhou (2003). The scale for entrepreneurial spirit was derived from Yujuico (2008). The scales have good reliability and validity (Table A1).

### Mediating variable: job crafting

Based on the 21-item job-crafting scale developed by Iida et al. (2021) and Du et al. (2018), this scale includes 15 items in three dimensions: increasing structural resources, social resources, and challenging demands (Table A1).

### Moderator variable: superiors’ developmental feedback

The scale came from Zhou (2003), and it contains three questions (Table A1).

### Control variables

First, this study controlled the relevant demographic variables, including gender, age, academic background, position, and years working. Then, company nature and industry, which might affect the empirical analysis, were controlled.

## Data analysis and results

### Data quality analysis

#### Reliability and validity analysis

As shown in Table 2, the α values of each variable ranged from 0.847 to 0.970 (all greater than 0.7), and the CR values ranged from 0.850 to 0.970 (all greater than 0.6), indicating a high level of internal consistency and CR. As shown in Table 2, the AVE values for each variable ranged from 0.654 to 0.788 (all greater than 0.5), indicating good convergent validity.

**TABLE 2:** Reliability and validity of the variables.

Variable name	Question item	Factor loading	α	CR	AVE
Innovation spirit (IS)	IS1	0.878	0.908	0.908	0.767
	IS2	0.876			
	IS3	0.873			
Sharing spirit (SS)	SS1	0.891	0.917	0.917	0.788
	SS2	0.917			
	SS3	0.854			
Practical spirit (PS)	PS1	0.915	0.912	0.913	0.724
	PS2	0.831			
	PS3	0.782			
	PS4	0.870			
Entrepreneurial spirit (ES)	ES1	0.867	0.911	0.912	0.722
	ES2	0.925			
	ES3	0.779			
	ES4	0.820			
Superiors’ developmental feedback (SDF)	SDF1	1.000	0.847	0.850	0.654
	SDF2	0.889			
	SDF3	0.872			
Job crafting (JC)	JC1	1.000	0.970	0.970	0.683
	JC2	0.921			
	JC3	0.969			
	JC4	0.998			
	JC5	0.960			
	JC6	0.915			
	JC7	0.998			
	JC8	0.974			
	JC9	0.944			
	JC10	0.939			
	JC11	0.915			
	JC12	0.974			
	JC13	0.927			
	JC14	0.895			
	JC15	0.947			
Knowledge workers’ innovation behavior (KWIB)	KWIB1	1.000	0.918	0.920	0.656
	KWIB2	1.132			
	KWIB3	1.055			
	KWIB4	0.885			
	KWIB5	0.928			
	KWIB6	1.020			

A secondary test of the measurement model was conducted using confirmatory factor analysis (Table 3). Compared to the other factor models, the 7-factor model had the best fit (χ^2^/df = 2.531, RMSEA = 0.059, NFI = 0.898, TLI = 0.929, IFI = 0.935, CFI = 0.935), again indicating good structural validity.

**TABLE 3:** Comparison of validation factors between measurement models.

Test quantity	χ ^2^/df	RMSEA	NFI	TLI	IFI	CFI
7-factor	2.531	0.059	0.898	0.929	0.935	0.935
6-factor	3.143	0.069	0.872	0.901	0.909	0.908
5-factor	4.693	0.091	0.807	0.829	0.841	0.841
4-factor	5.337	0.099	0.779	0.800	0.813	0.812
3-factor	6.146	0.108	0.744	0.762	0.777	0.776
2-factor	8.108	0.127	0.662	0.671	0.690	0.690
1-factor	9.897	0.142	0.586	0.589	0.612	0.611

DI, job crafting; CT, innovation spirit; IO, sharing spirit; FI, practical spirit; CO, entrepreneurial spirit; KIA, superiors’ developmental feedback; EIP, knowledge workers’ innovation behavior; 7-factor, CT, IO, FI, CO, KIA, EIP, DI; 6-factor, CT + IO, FI, CO, KIA, EIP, DI; 5-factor, CT + IO + FI, CO, KIA, EIP, DI; 4-factor, CT + IO + FI + CO, KIA, EIP, DI; 3-factor, CT + IO + FI + CO + KIA, EIP, DI; 2-factor, CT + IO + FI + CO + KIA + EIP, DI; 1-factor, CT + IO + FI + CO + KIA + EIP + DI.

#### Exploratory factor analysis

As shown in Table 4, the KMO test value is 0.958, Bartlett’s test of sphericity approximate Chi-square value is 15,394.662, and the probability of significance is 0.000, which indicates that it is suitable for factor analysis. The factors were extracted by principal component analysis and seven factors were found to have eigenvalues greater than 1. The proportion of variance explained by the seven factors was 47.059, 10.680, 5.083, 4.086, 3.598, 3.153, and 2.371%. The cumulative variance explained is 76.030%, which is more than 50%. Factor rotation by the maximum variance method showed that the factor loadings of each measurement question item were greater than 0.5. Corresponding to the variables of this study, Factor 1 represented job reinvention; Factor 2 represented employee innovative behavior; Factor 3 represented practical spirit; Factor 4 represented entrepreneurial spirit; Factor 5 represented innovative spirit; Factor 6 represented sharing spirit; and Factor 7 represented developmental feedback from superiors.

**TABLE 4:** Exploratory factor analysis results.

	Component							Common degree
	**1**	**2**	**3**	**4**	**5**	**6**	**7**	
IS1					0.764			0.851
IS2					0.733			0.825
IS3					0.765			0.840
SS1						0.749		0.861
SS2						0.724		0.874
SS3						0.736		0.834
PS1			0.838					0.852
PS2			0.787					0.769
PS3			0.806					0.753
PS4			0.792					0.816
ES1				0.746				0.790
ES2				0.805				0.845
ES3				0.801				0.757
ES4				0.834				0.799
KWIB1		0.723						0.697
KWIB2		0.674						0.722
KWIB3		0.746						0.740
KWIB4		0.740						0.726
KWIB5		0.720						0.704
KWIB6		0.797						0.779
JC1	0.806							0.801
JC2	0.793							0.734
JC3	0.780							0.733
JC4	0.792							0.708
JC5	0.776							0.702
JC6	0.811							0.746
JC7	0.705							0.658
JC8	0.768							0.665
JC9	0.779							0.715
JC10	0.812							0.742
JC11	0.781							0.743
JC12	0.772							0.710
JC13	0.806							0.742
JC14	0.722							0.612
JC15	0.802							0.761
SDF1							0.716	0.800
SDF2							0.734	0.733
SDF3							0.752	0.751
KMO value	0.958							
Bartlett’s test of sphericity	15394.662							
*p*-Value	0.000							
Eigenvalue	17.882	4.058	1.932	1.553	1.367	1.198	0.901	
Proportion of variance explained (%)	47.059	10.680	5.083	4.086	3.598	3.153	2.371	
Cumulative proportion of variance explained (%)	47.059	57.739	62.822	66.908	70.507	73.660	76.030	

The extraction method was principal component analysis. The rotation method is orthogonal rotation with Kaiser normalization. The rotation converges after seven iterations.

#### Common-method variance test

According to Hair et al. (2013), if the total variance explained by the first principal component is below the critical value of 50%, the degree of common-method bias is small. In this study, the total variance explained by the first principal component was 48%, indicating that the degree of common-method bias was small. As shown in Table 5, the change in each of the fit indices was less than 0.03 compared to the measurement model without the inclusion of the common factor. This indicates that the quality of fit of the measurement model including the common factor was not significantly improved, and there was no significant common-method bias in the measurements.

**TABLE 5:** Controlling unmeasured single latent variable model test results.

Test quantity	χ ^2^/df	RMSEA	NFI	TLI	IFI	CFI
There is no common factor	2.531	0.059	0.898	0.929	0.935	0.935
There are common factors	2.683	0.062	0.889	0.922	0.927	0.927

#### Correlation analysis

Pearson’s correlation was used to calculate the correlation coefficients between all variables to analyze the correlations between different variables (Table 6).

**TABLE 6:** Correlation analysis of variables.

Variable	Mean	SD	1	2	3	4	5	6	7
1. Innovation spirit	3.688	1.185	(0.876)						
2. Sharing spirit	3.670	1.116	0.664**	(0.887)					
3. Practical spirit	3.712	0.996	0.514**	0.553**	(0.851)				
4. Entrepreneurial spirit	3.525	1.158	0.522**	0.511**	0.408**	(0.850)			
5. Knowledge workers’ innovation behavior	3.526	0.932	0.621**	0.638**	0.555**	0.566**	(0.810)		
6. Job crafting	3.791	0.843	0.519**	0.522**	0.437**	0.479**	0.556**	(0.826)	
7. Superiors’ developmental feedback	3.748	0.860	0.365**	0.382**	0.372**	0.341**	0.369**	0.655**	(0.809)

The symbol “**” indicates *p* < 0.01 (two-sided test), and data in parentheses are the AVE square root.

As shown in Table 6, the means of all variables were within a reasonable range, and the standard deviations were all greater than 0.8, indicating that the data could be used to analyze the differences between different variables in the model. In this article, we also performed variance inflation factor tests. The results showed that the VIF value of each variable was less than 3, which is far below the critical value of 10, indicating that there was no multicollinearity in the data.

### Hypothesis test

#### Direct effect test

This study used multiple linear regression to test the utility of the effects of innovation spirit, sharing spirit, practical spirit, and entrepreneurial spirit on job crafting and the utility of the effect of job crafting on knowledge workers’ innovative behavior. As shown in Table 7, M1 tested the effect of the control variables on job crafting, and M2 added innovation spirit to M1 to verify the direct effect of innovation spirit on job crafting. The test results showed that there was a significant positive effect of innovation spirit on job crafting (β = 0.356, *p* < 0.001); thus, H2a was verified. M3 added sharing spirit to M1 to test the effect of sharing spirit on job crafting. The results showed that sharing spirit had a significant positive effect on job crafting (β = 0.384, *p* < 0.001); thus, H2b was verified. M4 was added to M1 to test the direct effect of practical spirit on job crafting. The results showed that practical spirit had a significant positive effect on job crafting (β = 0.353, *p* < 0.001); thus, H2c was verified. M5 added entrepreneurial spirit to M1 to test the direct effect of entrepreneurial spirit on job crafting. The results showed a significant positive effect of entrepreneurial spirit on job crafting (β = 0.336, *p* < 0.001), supporting H2d. M6 tested the effect of the control variables on knowledge workers’ innovative behavior, and M7 added job crafting to M6 to test the direct effect of job crafting on knowledge workers’ innovative behavior. The results showed a significant positive effect of job crafting on knowledge workers’ innovative behavior (β = 0.585, *p* < 0.001); thus, H3 was verified.

**TABLE 7:** Results of the regression analysis of direct effects.

Variable	Job crafting						Knowledge workers’ innovative behavior
	M1	M2	M 3	M4	M5	M6	M7
Innovation spirit		0.356***					
Sharing spirit			0.384***				
Practical spirit				0.353***			
Entrepreneurial spirit					0.336***		
Job Crafting							0.585***
Gender	-0.014	-0.028	0.001	-0.045	0.027	0.118	0.126
Age	-0.143**	-0.089	-0.111*	-0.096	-0.118*	-0.219***	-0.135**
Education	0.111	0.060	0.052	0.077	0.060	0.250**	0.186*
Working years	0.127*	0.094	0.085	0.094	0.095	0.186**	0.111*
Position	0.070	0.037	0.080*	0.044	0.036	0.077	0.036
Ownership	-0.054*	-0.041	-0.037	-0.045	-0.046*	-0.038	-0.007
Industry	0.051	0.039	0.024	0.038	0.036	0.028	-0.002
*R* ^2^	0.045	0.290	0.298	0.215	0.255	0.072	0.340
Adjusted *R*^2^	0.030	0.277	0.285	0.201	0.243	0.057	0.328
*ΔR* ^2^	0.045	0.245	0.253	0.170	0.209	0.072	0.267
*F* value	2.935**	22.258***	23.120***	14.938***	20.798***	4.856***	28.034***
*ΔF*	2.935**	150.492***	157.079***	94.562***	136.324***	4.856***	176.617***

The symbols “*, **, and ***” represent *p* < 0.05, *p* < 0.01, and *p* < 0.001, respectively.

#### Mediating utility test

According to Siren et al. (2016), mediating utility must satisfy three conditions: (1) the independent variable is significantly correlated with the dependent variable, (2) the independent variable is significantly correlated with the mediating variable, and (3) when the mediating variable is put into the regression equation, it is significantly correlated with the dependent variable, the correlation between the independent variable and the dependent variable is significantly weakened as partial mediation, and the correlation between the independent variable and the dependent variable is not significant as full mediation.

This study conducted a mediating utility analysis based on the above. Intermediary path 1 (innovation spirit → job crafting → knowledge workers’ innovation behavior) was tested, as shown in Table 8. M8 added innovation spirit to M6. The results showed that innovation spirit had a significant positive effect on knowledge workers’ innovation behavior (β = 0.468, *p* < 0.001); thus, H1a was verified and condition 1 of mediating utility was satisfied. The significant positive effect of innovation spirit on job crafting was verified above in the test of H2a, thus satisfying condition 2 of mediating utility. When job crafting was added to the M12 regression equation, it had a significant positive effect on knowledge workers’ innovative behavior (β = 0.333, *p* < 0.001). The relationship between innovation spirit and knowledge workers’ innovative behavior was significant but significantly weaker (β = 0.350, *p* < 0.001), satisfying condition 3. The above verifications fully indicate that job crafting played a partial mediating role between innovation spirit and knowledge workers’ innovation behavior, verifying H4a.

**TABLE 8:** Results of the regression analysis of mediation effects.

Variable	Knowledge workers’ innovative behavior									
	M6	M7	M8	M9	M10	M11	M12	M13	M14	M15
Innovation spirit			0.468***				0.350***			
Sharing spirit				0.523***				0.406***		
Practical spirit					0.494***				0.350***	
Entrepreneurial spirit						0.441***				0.313***
Job crafting		0.585***					0.333***	0.305***	0.409***	0.382***
Gender	0.118	0.126	0.100	0.138*	0.075	0.171*	0.109	0.138*	0.093	0.161*
Age	-0.219***	-0.135**	-0.148**	-0.175***	-0.152**	-0.186***	-0.118**	-0.141**	-0.113*	-0.141**
Education	0.250**	0.186*	0.184**	0.171*	0.203**	0.184*	0.164*	0.155*	0.172*	0.161*
Working years	0.186**	0.111*	0.142**	0.128**	0.140**	0.143**	0.111*	0.102*	0.101*	0.107*
Position	0.077	0.036	0.034	0.091*	0.042	0.032	0.022	0.067	0.023	0.019
Ownership	-0.038	-0.007	-0.021	-0.015	-0.025	-0.028	-0.008	-0.004	-0.007	-0.010
Industry	0.028	-0.002	0.013	-0.009	0.010	0.008	0.000	-0.016	-0.006	-0.005
*R* ^2^	0.072	0.340	0.419	0.457	0.344	0.335	0.483	0.510	0.452	0.455
Adjusted *R*^2^	0.057	0.328	0.408	0.447	0.332	0.324	0.472	0.500	0.440	0.444
*ΔR* ^2^	0.072	0.267	0.346	0.385	0.272	0.274	0.411	0.438	0.379	0.383
*F* value	4.856***	28.034***	39.239***	45.830***	28.627***	30.536***	45.180***	50.333***	39.813***	40.385***
*ΔF*	4.856***	176.617***	259.791***	308.712***	181.021***	199.925***	172.942***	194.457***	150.531***	152.922***

The symbols “*, **, and ***” represent *p* < 0.05, *p* < 0.01, and *p* < 0.001, respectively.

Intermediary path 2 (sharing spirit → job crafting → knowledge workers’ innovation behavior) was tested. As shown in Table 8, M9 added sharing spirit to M6. The results showed that sharing spirit had a significant positive effect on knowledge workers’ innovation behavior (β = 0.523, *p* < 0.001); thus, H1b was verified and condition 1 was satisfied. Condition 1 was also satisfied since sharing spirit had a significant positive effect on job crafting, as verified in the above test of H2b. When job crafting was added to the M13 regression equation, it had a significant positive effect on knowledge workers’ innovation behavior (β = 0.305, *p* < 0.001). The relationship between sharing spirit and knowledge workers’ innovation behavior was significant but significantly weaker (β = 0.406, *p* < 0.001), satisfying condition 3. The above validation also indicates that job crafting played a partial mediating role between sharing spirit and knowledge workers’ innovation behavior; thus, H4b was verified.

Intermediary path 3 (practical spirit → job crafting → knowledge workers’ innovation behavior) was tested. As shown in Table 8, M10 added practical spirit to M6. The results showed that practical spirit had a significant positive effect on knowledge workers’ innovation behavior (β = 0.494, *p* < 0.001); thus, H1c was verified and condition 1 was satisfied. The significant positive effect of practical spirit on job crafting was verified in the test of H2c, satisfying condition 2. When job crafting was added to the M14 regression equation, it had a significant positive effect on knowledge workers’ innovative behavior (β = 0.409, *p* < 0.001). The relationship between practical spirit and knowledge workers’ innovative behavior was significant but significantly weaker (β = 0.350, *p* < 0.001); thus, condition 3 was satisfied. The above validation results indicate that job crafting played a partial mediating role between practical spirit and knowledge workers’ innovation behavior; thus, H4c was verified.

Intermediary path 4 (entrepreneurial → job crafting → knowledge workers’ innovation behavior) was tested. As shown in Table 8, M11 added entrepreneurial spirit to M6. The results showed that entrepreneurial spirit had a significant positive effect on knowledge workers’ innovation behavior (β = 0.441, *p* < 0.001); thus, H1d was verified and condition 1 was satisfied. Entrepreneurial spirit had a significant positive effect on job crafting, which was verified in the test of H2d; thus, condition 2 was satisfied. When job crafting was added to the M15 regression equation, it had a significant positive effect on knowledge workers’ innovative behavior (β = 0.382, *p* < 0.001). The relationship between entrepreneurial spirit and knowledge workers’ innovative behavior was significant but significantly weaker (β = 0.313, *p* < 0.001), satisfying condition 3. The above validation results indicate that job crafting played a partial mediating role between entrepreneurial spirit and knowledge workers’ innovation behavior; thus, H4d was verified.

#### Moderating effect test

Table 9 shows the results for the moderating effect of superiors’ developmental feedback. M16 added superiors’ developmental feedback to M2. The results showed that superiors’ developmental feedback (β = 0.521, *p* < 0.001) had a significant positive effect on job crafting. M17 added superiors’ developmental feedback to M3. The results showed that superiors’ developmental feedback (β = 0.513, *p* < 0.001) had a significant positive effect on job crafting. M18 added superiors’ developmental feedback to M4, and the results showed that superiors’ developmental feedback (β = 0.554, *p* < 0.001) had a significant positive effect on job crafting. M19 added superiors’ developmental feedback to M5, and the results showed that superiors’ developmental feedback (β = 0.539, *p* < 0.001) had a significant positive effect on job crafting. M20 added an interaction term for superiors’ developmental feedback and innovation spirit to M16. The results showed that the interaction term for superiors’ developmental feedback and innovation spirit (β = 0.132, *p* < 0.001) was significantly positive; thus, H5a was verified. M21 added an interaction term for superiors’ developmental feedback and sharing spirit to M17. The results showed that the interaction term for superiors’ developmental feedback and sharing spirit (β = 0.123, *p* < 0.001) was significantly positive; thus, H5b was verified. M22 added an interaction term for superiors’ developmental feedback and practical spirit to M18. The results showed that the interaction term of superiors’ developmental feedback and practical spirit (β = 0.161, *p* < 0.001) was significantly positive; thus, H5c was verified. M23 added the interaction term of superiors’ developmental feedback with entrepreneurial spirit to M19. The results indicated that the interaction term of superiors’ developmental feedback with entrepreneurial spirit (β = 0.064, *p* < 0.05) was significantly positive, thus H5d was verified.

**TABLE 9:** Results of the regression analysis of moderating effects.

Variable	Job crafting								
	M1	M16	M17	M18	M19	M20	M21	M22	M23
Innovation spirit		0.223***				0.261**			
Sharing spirit			0.233***				0.272***		
Practical spirit				0.179***				0.247***	
Entrepreneurial spirit					0.205***				0.221***
Superiors’ developmental feedback		0.521***	0.513***	0.554***	0.539***	0.548***	0.536***	0.606***	0.550***
Innovation spirit × superiors’ developmental feedback						0.132***			
Sharing spirit × superiors’ developmental feedback							0.123***		
Practical spirit × superiors’ developmental feedback								0.161***	
Entrepreneurial spirit × superiors’ developmental feedback									0.064*
Gender	-0.014	0.056	0.073	0.054	0.093	0.069	0.084	0.056	0.102
Age	-0.143**	-0.063	-0.079*	-0.070	-0.080*	-0.064	-0.086*	-0.038	-0.083*
Education	0.111	0.021	0.018	0.032	0.020	0.009	0.009	0.018	0.014
Working years	0.127*	0.101*	0.096*	0.105*	0.102*	0.111**	0.107**	0.087*	0.106*
Position	0.070	0.010	0.037	0.015	0.008	-0.002	0.032	0.001	0.003
Ownership	-0.054*	-0.027	-0.025	-0.029	-0.029	-0.022	-0.021	-0.022	-0.025
Industry	0.051	0.040*	0.031	0.041*	0.038*	0.034	0.023	0.036	0.034
*R* ^2^	0.045	0.528	0.523	0.482	0.513	0.553	0.544	0.515	0.519
Adjusted *R*^2^	0.030	0.518	0.514	0.471	0.503	0.543	0.534	0.504	0.508
*ΔR* ^2^	0.045	0.483	0.479	0.437	0.468	0.508	0.500	0.470	0.474
*F* value	2.935**	54.102***	53.095***	44.912***	50.932***	53.731***	51.869***	46.128***	46.873***
*ΔF*	2.935**	222.765***	218.434***	183.266***	209.141***	164.569***	158.640***	140.363***	142.734***

The symbols “*, **, and ***” represent *p* < 0.05, *p* < 0.01, and *p* < 0.001, respectively.

**FIGURE 2:**
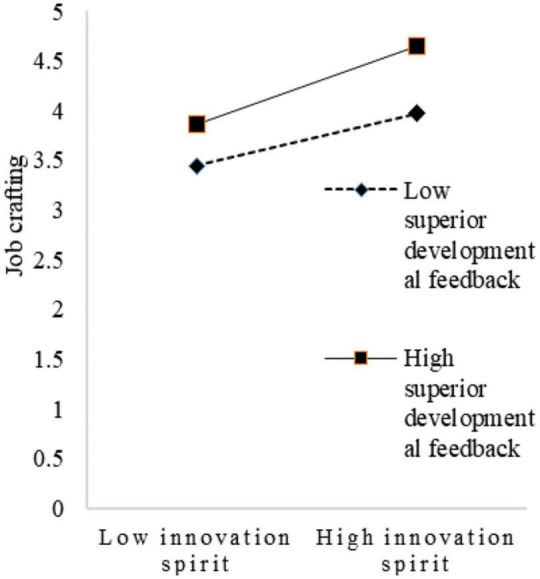
Moderating role in innovation spirit.

**FIGURE 3:**
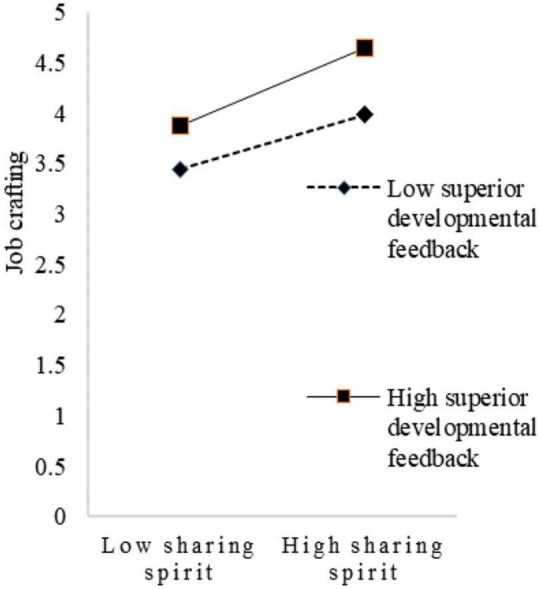
Moderating role in sharing spirit.

**FIGURE 4:**
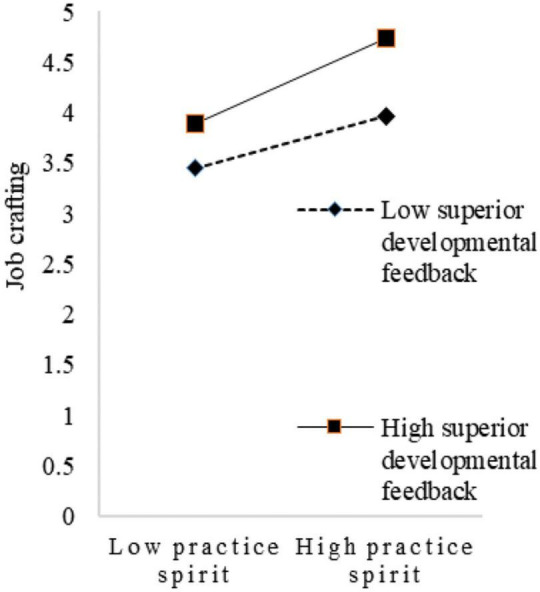
Moderating role in practice spirit.

**FIGURE 5:**
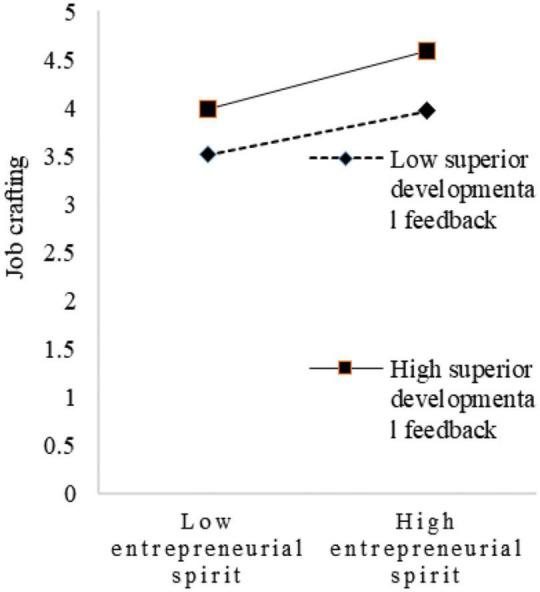
Moderating role in entrepreneurial spirit.

To visualize the moderating effect of superiors’ developmental feedback, this study set two scenarios for the variable, with one standard deviation above and one below the mean for simple slope mapping. As shown in Figure 2, when superiors’ developmental feedback is low, the trend of the effect of innovation spirit on job crafting is relatively flat. When superiors’ developmental feedback is high, the trend of the effect of innovation spirit on job crafting becomes relatively steep. Thus, superiors’ developmental feedback had a positive moderating effect on the relationship between innovation spirit and job crafting. As shown in Figure 3, when superiors’ developmental feedback is low, the trend of the influence of sharing spirit on job crafting is relatively flat. When superiors’ developmental feedback is high, the trend of the influence of sharing spirit on job crafting is relatively steep. Therefore, superiors’ developmental feedback had a positive moderating effect on the relationship between sharing spirit and job crafting. Similarly, as shown in Figure 4, superiors’ developmental feedback is high, the trend of the influence of practical spirit on job crafting is relatively steep. Therefore, superiors’ developmental feedback had a positive moderating effect on the relationship between practical spirit and job crafting. Similarly, as shown in Figure 5, superiors’ developmental feedback had a positive moderating effect on the relationship between entrepreneurial spirit and job crafting.

#### Moderated mediating effect test

Table 10 shows the results for the moderated mediating t of superiors’ developmental feedback. As shown in Table 10, when the superiors’ developmental feedback is low, the indirect effect of innovative spirit on innovative behavior through job crafting is 0.0532, with a standard error of 0.0153, 95% confidence interval of [0.0243, 0.0840], which does not contain 0, indicating a significant indirect effect. When the superiors’ developmental feedback is high, the indirect effect of innovative spirit on innovative behavior through job crafting is 0.1315, with a standard error of 0.0345, 95% confidence interval of [0.0664, 0.2023], not including 0, indicating a significant indirect effect. The difference in the indirect effect of supervisors’ developmental feedback in the higher than low condition was 0.0783, with a standard error of 0.0293, 95% confidence interval of [0.0237, 0.1395], and did not contain 0, indicating that the difference in the indirect effect was significant, and that the mediating role of the moderated existed, thus H6a was proven. Similarly, H6b, H6c, and H6d were verified.

**TABLE 10:** Results of moderated mediating effect tests.

Intermediate variable	Moderator variable: superiors’ developmental feedback	Implicit variable: innovative behavior			
		Effect value	Standard error	95% confidence interval	
				Min	Max
Innovation spirit					
Job crafting	Low value	0.0532	0.0153	0.0243	0.0840
	High value	0.1315	0.0345	0.0664	0.2023
	D-value	0.0783	0.0293	0.0237	0.1395
Sharing spirit					
Job crafting	Low value	0.0553	0.0165	0.0248	0.0889
	High value	0.1219	0.0345	0.0594	0.1925
	D-value	0.0666	0.0282	0.0145	0.1256
Practical spirit					
Job crafting	Low value	0.0480	0.0216	0.0097	0.0936
	High value	0.1681	0.0518	0.0728	0.2777
	D-value	0.1201	0.0433	0.0380	0.2088
Entrepreneurial spirit					
Job crafting	Low value	0.0675	0.0181	0.0337	0.1041
	High value	0.1112	0.0354	0.0465	0.1852
	D-value	0.0437	0.0309	0.0152	0.1064

## Discussion

### Discussion of main findings

(1) Innovation spirit, sharing spirit, practical spirit, and entrepreneurial spirit all positively affected knowledge workers’ innovative behavior (H1a–H1d were supported). This indicates that the maker spirit plays an important role in stimulating the innovative behavior of knowledge workers. This result is consistent with the existing literature on the effects of innovation, practice, sharing, entrepreneurship, and innovation behavior (Yuan, 2007; Luo et al., 2010; Shi, 2012; Cui and Yu, 2021). For this reason, companies and managers should focus on promoting the maker spirit among employees, especially knowledge workers, and improve the construction of maker spaces to stimulate the leading effect of the spirit of creativity. enterprises and managers can establish the innovation spirit by improving the organizational structure of innovation and constructing the “innovative fault-tolerant mechanism”; they can cultivate the sharing spirit by breaking the traditional closed innovation model and shaping the value of collective collaboration; they can strengthen the practical spirit by advocating practical problem solving and emphasizing applied innovation; they can stimulate the entrepreneurial spirit by creating an entrepreneurial climate and promoting organizational empowerment. From the above four dimensions, they can deepen the maker spirit of knowledge employees and promote their innovative behavior.

(2) Innovation spirit, sharing spirit, practical spirit, and entrepreneurial spirit all positively affected job crafting (H2a–H2d were supported). This indicates that when knowledge workers are more innovative, they are more likely to transform existing resources in ways that are useful for them, thus facilitating their job crafting. When knowledge workers are more conscious of sharing, they are better able to access resources to cope with ever-changing environments and improve their job crafting. The more hands-on knowledge workers are, the more they are able to seek out resources before others do, and the more they are able to seize opportunities to find internal resources that match their work tasks, thus facilitating job crafting. When knowledge workers have higher entrepreneurship awareness, they are better equipped to redesign their work tasks through novel adaptations. This finding confirms that knowledge workers can undertake job crafting by increasing their own entrepreneurial spirit. For this reason, companies and managers should adopt the maker spirit as a basis to promote the initiative of knowledge workers to carry out job crafting and improve their comprehensive quality through the leading role of innovation, sharing, practice, and entrepreneurship.

(3) Job crafting positively affected knowledge workers’ innovative behavior (H3 holds). This result is consistent with the findings of the existing related literature (Taa et al., 2020; Xu and Zhao, 2020; Chun, 2021; Huang et al., 2021). It means that when knowledge workers have a stronger ability to craft their work, they are better able to use their strengths to adapt to dynamic environments, thus becoming more creative and supporting the smooth implementation of their innovative behavior. This finding confirms that knowledge workers can positively affect their innovative behavior by improving their job-crafting skills. For this reason, enterprises and managers should attach importance to knowledge workers’ job crafting behavior, and provide complete resources, environment and atmosphere guarantee for the job crafting of knowledge workers while stimulating their motivation for job crafting.

(4) Job crafting mediated the relationship between innovation spirit and knowledge workers’ innovative behavior, between sharing spirit and knowledge workers’ innovative behavior, between practical spirit and knowledge workers’ innovative behavior, and between entrepreneurial spirit and knowledge workers’ innovative behavior (verifying H4a–H4d). This suggests that job crafting can promote creativity and thus knowledge workers’ innovative behavior. Existing literature on the mediating role of job crafting in the relationship between maker spirit and innovative behavior is little. Similar literature mainly explored the mediating effects of job crafting on leadership characteristics and employee knowledge sharing (Park et al., 2015), employee initiative and employee adaptability (Le, 2020), leadership style and employee innovation performance (Alshura et al., 2023), leadership personality and employee happiness (Yang et al., 2012), and focus regulation and employee job engagement (Yang et al., 2012). The results of the above literature all suggest that job crafting plays an important mediating role in influencing employee behavior, which also provides a degree of theoretical support for the findings of this article. Based on the mediating effect of job crafting, companies and managers should take job crafting as the focus to give full play to the bridging role of it, so as to contribute to the implementation of innovative activities by knowledge workers.

(5) Superiors’ developmental feedback had a positive moderating effect between innovation spirit and job crafting, between sharing spirit and job crafting, between practical spirit and job crafting, and between entrepreneurial spirit and job crafting (H5a–H5d were supported). To the best of our knowledge, no study has explored superiors’ developmental feedback as a moderating role in the relationship between maker spirit and job crafting. Similar literature has mainly explored the direct effect of superiors’ developmental feedback on employee growth (Redling, 2010), the mechanism by which superiors’ developmental feedback affects employees’ proactive change behavior (Miao and Shu, 2021), the effect of superiors’ developmental feedback on employees’ innovative behavior (Su et al., 2019; Jiang et al., 2022), and the moderating effect of superiors’ developmental feedback on the influence of obstructive pressure on employees’ creativity (Sun et al., 2019). As such, the study contributes to superiors’ developmental feedback literature, and enhances the theoretical perspective that superiors’ developmental feedback can moderate the influence of maker spirit on job crafting Based on it, enterprises and managers should firstly pay full attention to the role of superiors’ developmental feedback in promoting knowledge workers’ innovative behavior, and focus on improving the cognition of superiors’ developmental feedback. Secondly, the superiors’ developmental feedback such as positive evaluation, inclusive attitude, sincere concern should be adopted to promote the growth of employees, encourage employees to actively cultivate the spirit of maker and carry out work remodeling, so that employees can break the conventional thinking, generate creative inspiration, and actively adopt innovative behaviors.

### Theoretical significance

Existing studies of the maker spirit suffer from overly vague and broad definitions of the concept, and few scholars have integrated the maker and innovative behavior into a unified analytical framework to comprehensively analyzed the influence mechanism of maker spirit on innovation behavior. To fill this research gap, this article constructed an empirical model based on the introduction of variables such as job crafting and superiors’ developmental feedback to investigate the impact of maker spirit on the knowledge workers’ innovative behavior. The main contributions of this article are as follows:

(1) Taking the maker spirit as the core, this article explains the connotation of the maker spirit and its relationship with the innovative behavior of knowledge workers. This enriches theoretical research in the field of maker spirit.
(2) This study clarifies the influence mechanism of maker spirit on knowledge workers’ innovative behavior, and provides a theoretical basis for the state or relevant managers to stimulate the innovative behavior of knowledge workers.

### Practical implications

The practical contributions of this study are as follows:

(1) The findings indicate that knowledge workers’ innovation behavior can be enhanced by cultivating their maker spirit, thereby achieving the goal of improving knowledge-worker productivity.
(2) The findings can provide a basis for further promoting innovation and entrepreneurship in China in the era of mass entrepreneurship, further contributing to cultural and economic development.
(3) This study can provide a basis for decision-making to further enhance the innovative behavior of knowledge workers in knowledge-intensive industries.

## Limitations and future work

Due to the complexity of the research problems and the limited capacity of the authors, there are still some research limitations in this study. To address these limitations, we will use this as a direction and further refine it in subsequent research. Specifically, the details are as follows:

(1) The data were obtained using questionnaires. As a result, there are limitations related to elements such as the narrow sample area. The connotation and extension of knowledge workers should be further studied in the process of questionnaire distribution to improve its relevance. The strong subjectivity of the questionnaire distribution and measurement indicators to some extent affected the accuracy of the findings. In future research, therefore, more effort should be made in terms of sample collection and use, as well as the timing of distribution.
(2) This study only discusses the influence of maker spirit on knowledge workers’ innovative behavior from the perspective of static development, but in fact, this should be a dynamic development process. Therefore, in the future, we will improve the research model to study the relationship between the two from a dynamic perspective.
(3) The paper lacks a comprehensive and systematic theoretical explanation on the effects of maker spirit on knowledge workers’ innovation behavior, the mediating effect of the job crafting, and the moderating effect of superiors’ developmental feedback. This makes the theoretical basis of the paper relatively weak and reduces the stability and persuasiveness of the empirical results. In future work, on the basis of a comprehensive review of the existing literature, we will integrate relevant theories and improve the theoretical exposition of this article, so as to provide strong theoretical support for the research.

## Data availability statement

The original contributions presented in this study are included in this article/supplementary material, further inquiries can be directed to the corresponding author.

## Ethics statement

This study was carried out in accordance with the recommendations of Ethics Committee of Hohai University and Jiangsu University of Science and Technology with written informed consent from all subjects in accordance with the Declaration of Helsinki. The protocol was approved by the Ethics Committee of Hohai University and Jiangsu University of Science and Technology.

## Author contributions

QX led the research design, data analysis, and drafted this manuscript. CL guided the research design and revised the manuscript substantially. MZ and HJ made contributions in data analysis and manuscript revision. All authors approved the final version.

## Appendix A

**TABLE A1:** Measurement scale.

Variable	No.	Question item
Knowledge workers’ innovation behavior (KWIB)	KWIB1	At work, I try to use new methods, new technologies, or new programs with problems.
	KWIB2	I often come up with creative ideas at work.
	KWIB3	I often communicate with others and suggest my own new ideas.
	KWIB4	To realize my creative ideas, I find ways to get the resources I need.
	KWIB5	To implement my creative ideas, I actively develop appropriate plans.
	KWIB6	Overall, I am an innovative person.
Innovation spirit (IS)	IS1	I always have a steady stream of ideas.
	IS2	I like using innovative means to solve problems.
	IS3	I emphasize the degree of innovation in product design.
Sharing spirit (SS)	SS1	I am willing to adopt valuable new perspectives offered by executive team members.
	SS2	I possess and am willing to share new decision-making knowledge.
	SS3	I have new views about the issues discussed and am willing to actively share them with you.
Practical spirit (PS)	PS1	I seize market opportunities before my peer competitors.
	PS2	I can remain sensitive to dynamic changes in the external environment.
	PS3	I value the development of market opportunities more than my peer competitors.
	PS4	I have a new perspective on the issues discussed and am willing to actively share my thoughts with others.
Entrepreneurial spirit (ES)	ES1	I attach great importance to unique design in the production process and its mode.
	ES2	I would prefer to choose solutions with high risks and high returns in strategic decision-making.
	ES3	In the face of favorable market opportunities, I will actively take appropriate strategies.
	ES4	Faced with emerging target markets, our company is usually the pioneer among many peers.
Job crafting (JC)	JC1	I try to improve my abilities.
	JC2	I try to make myself more professional.
	JC3	I try to acquire new knowledge from my work.
	JC4	I give full play to my abilities.
	JC5	I decide the way I do things.
	JC6	I ask my superiors for guidance and help.
	JC7	I will ask my supervisor if they are satisfied with my work.
	JC8	I hope to get encouragement from my superiors.
	JC9	I ask others what they think about their job performance.
	JC10	I seek the advice from my colleagues.
	JC11	When I am interested in a project, I volunteer to join it.
	JC12	I am the first person to try out new things.
	JC13	I view the off-season for my work as a preparation period for new projects.
	JC14	I am willing to undertake extra work without the extra payment.
	JC15	I seek new challenges by examining relationships across potential aspects of the work.
Superiors’ developmental feedback (SDF)	SDF1	My immediate superior gives me feedback mainly to help me learn and improve.
	SDF2	My immediate superior has never provided information that supports my work and growth.
	SDF3	My immediate superiors give me valuable information about how to improve my work performance.
